# Supplementary material for: Multiscale domain identification for spatial transcriptomics via persistent homology
Source: Cell Rep Methods. 2026 Mar 30;6(5):101376. doi: 10.1016/j.crmeth.2026.101376 (PMC13198003; doi:10.1016/j.crmeth.2026.101376)
Supplement: Document S1. Figures S1−S3, Table S1, and Methods S1 and S2 [file mmc1.pdf]

Cell Reports Methods, Volume *6*

Supplemental information

Multiscale domain identification for spatial transcriptomics via persistent homology

Perry Beamer and Zixuan Cang

Supplementary Material

Table S1

Table S1: Glossary of Relevant Technical Terms in the Main Text, related to Figure 1.

Term	Definition
Region	A general term referring to a neighborhood or area in a tissue
Domain	An internally homogeneous region corresponding to morphological structure
Spot	A single annotated transcript
Scale	A general term that describes the size of domains in a clustering algorithm.
Scale parameter, k	A hyperparameter that controls the scale of output in the Leiden clustering algorithm
Graph	A structure consisting of nodes and edges, connections between nodes
Clustering filtration	A weighted graph where nodes represent domains at different scales and where edges represent connections between nodes. Edges are weighted by the strength of the connection between domains

Figure S1

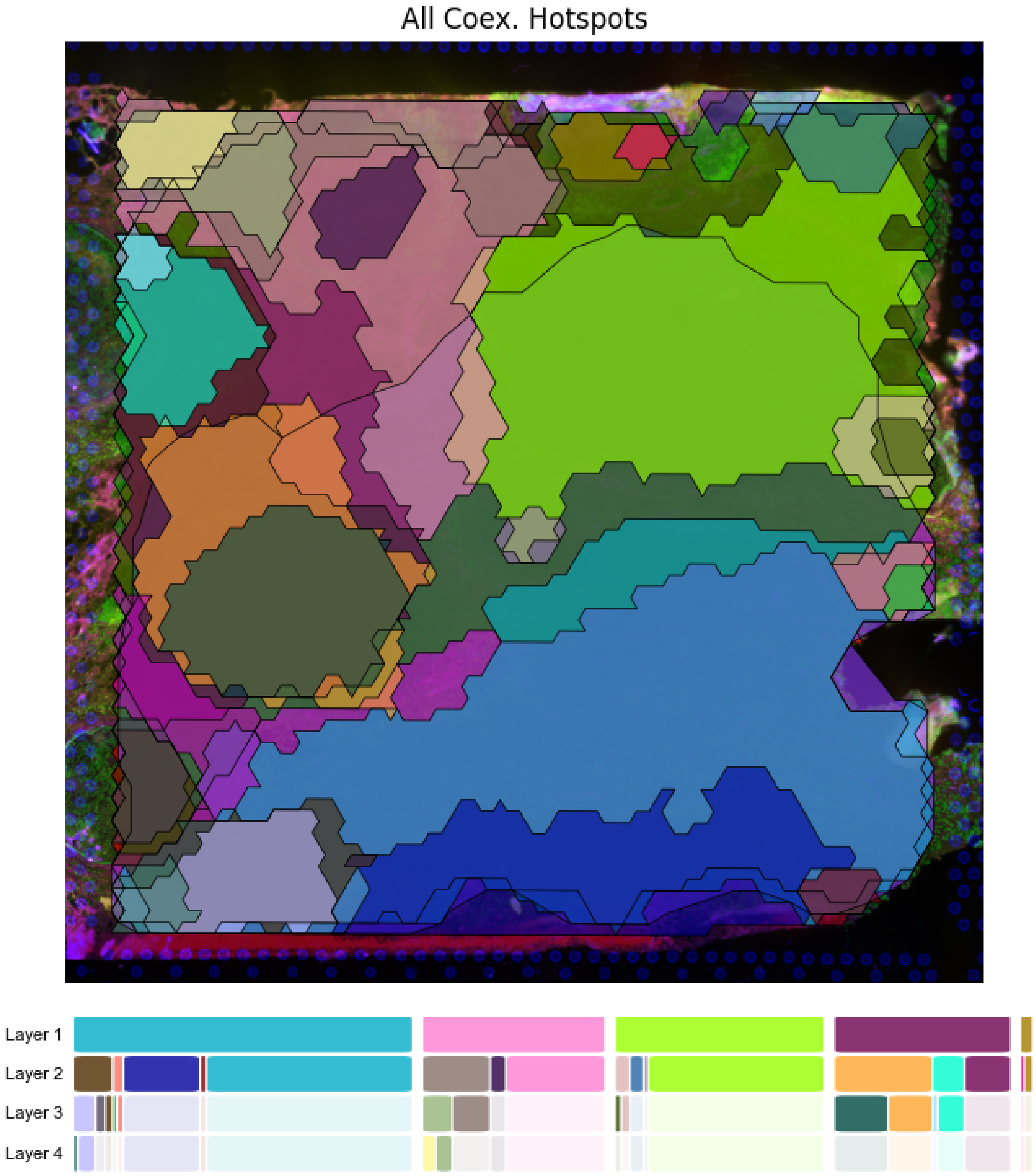

Figure S1: NeST results for IDC dataset, related to Figure 3.

Figure S2

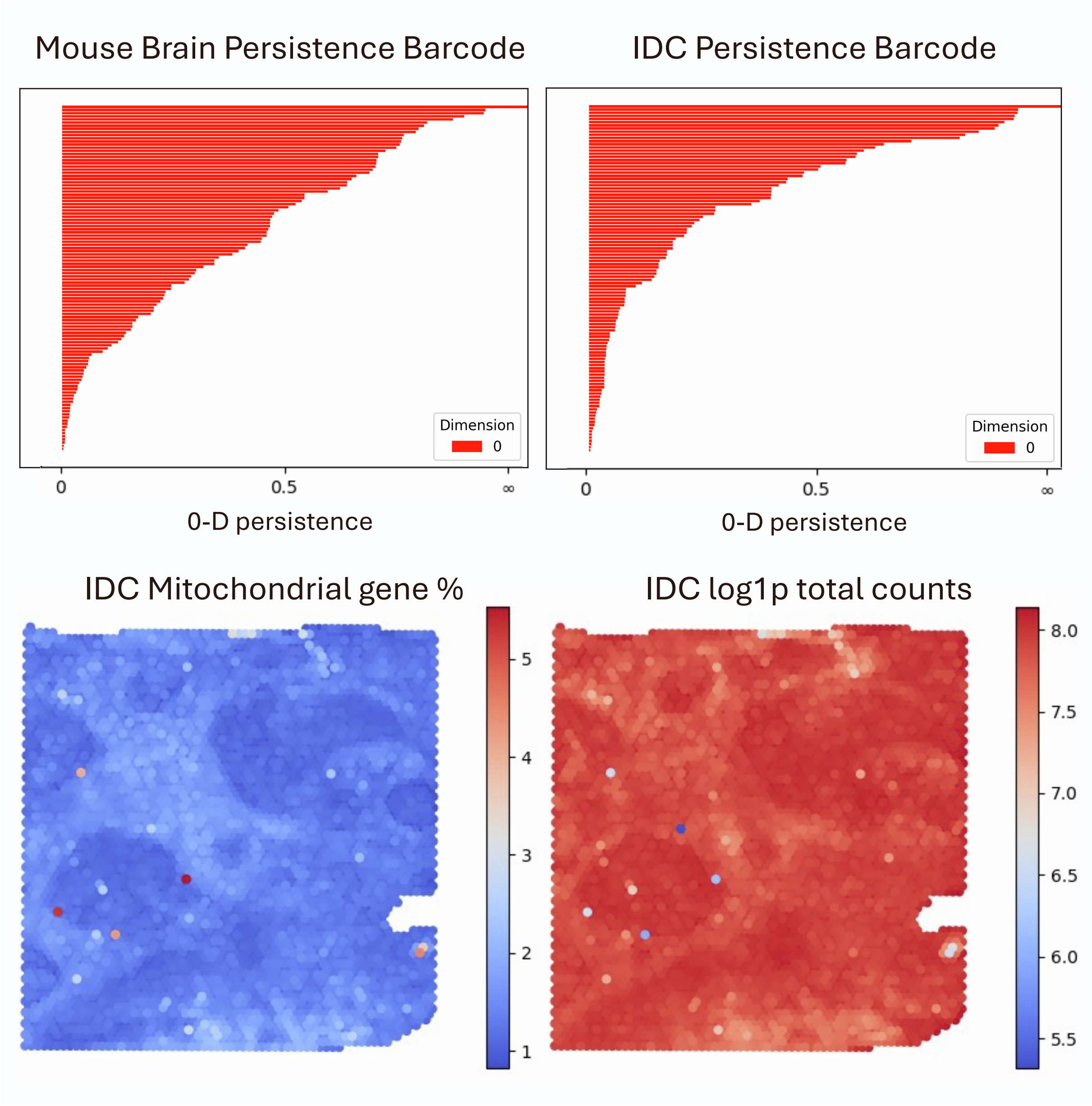

Figure S2: Persistence diagrams for the mouse brain and IDC datasets and quality control (QC) metrics metrics for IDC dataset, related to Figures 2 and 3.
Top: 0-D persistence diagrams from the cluster filtration for mouse brain and IDC datasets, *Bottom*: QC metrics (% mitochondrial genes and log1p total counts) for IDC dataset.

Figure S3

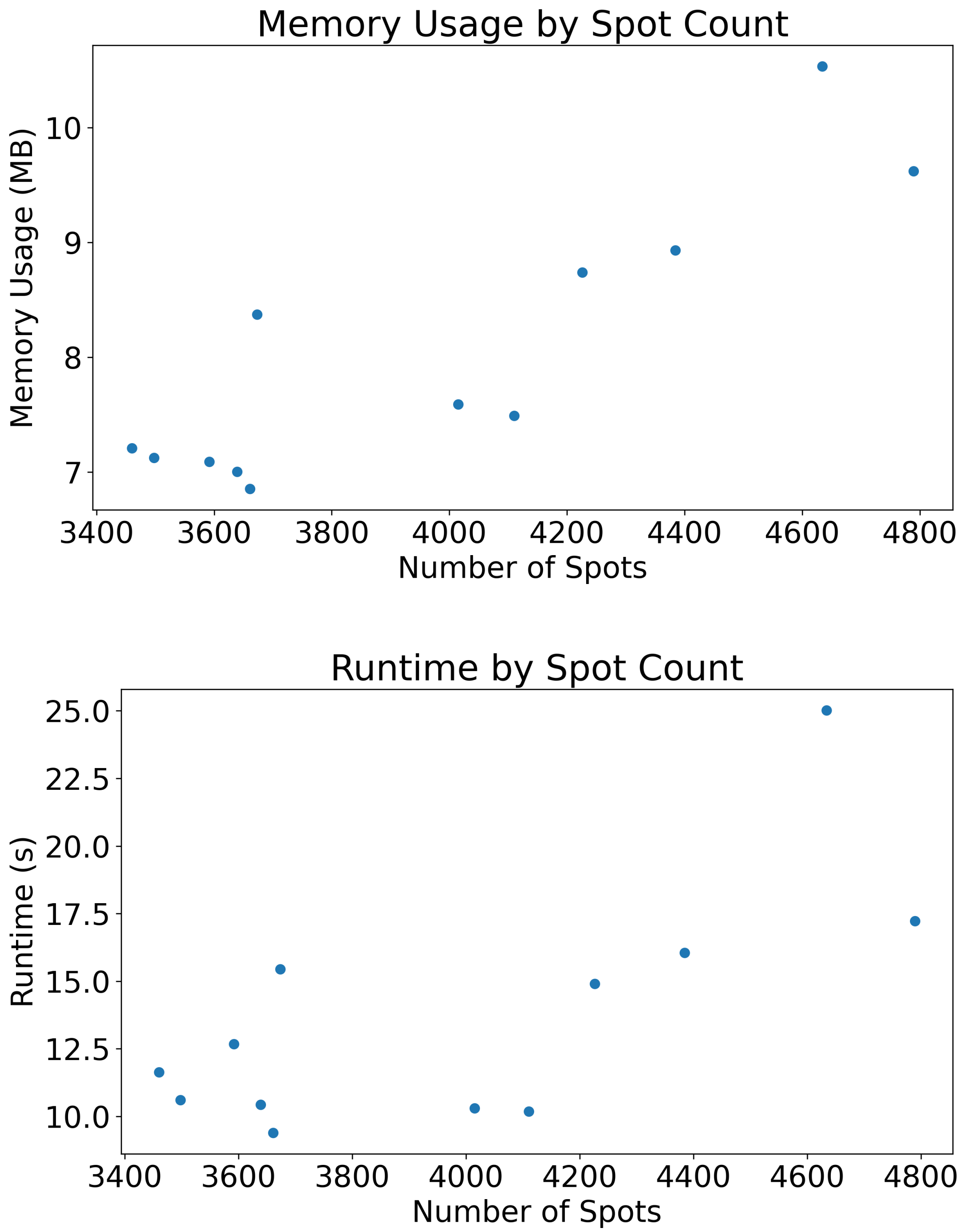

Figure S3: Memory and runtime versus spot count across human dorsolateral prefrontal cortex (DLPFC) datasets, related to Figure 4.
Top: Runtime (s) by spot count for DLPFC data, *Bottom*: Memory usage (MB) by spot count for DLPFC data.

Methods S1: Basic formulation of persistent homology, related to STAR Methods.

In this section, we will define the basic terms and constructions of persistent homology. A thorough exposition of these and other fundamental definitions in topology is available in [S1] and [S2]. Further background on persistent homology can be found in [S3].

Definition 1. *Given a non-empty vertex set V, a **simplicial complex** K is a collection of non-empty subsets of V, whose elements σ are called **simplices**. A simplicial complex must also satisfy the following properties:*

$$v \in V \implies \{v\} \in K \tag{1}$$

$$\tau \subset \sigma \text{ and } \sigma \in K \implies \tau \in K \tag{2}$$

Definition 2. *Given a simplicial complex K we will denote the **n-th chain group of $\mathbf{K}$ with coefficients in** Abelian group G by*

$$\mathbf{C}_n(K;G) := \left\{ \sum_{\sigma} x_\sigma \sigma : x_\sigma \in G, \sigma \in K, \dim \sigma = n \right\}$$

Definition 3. *The **boundary map** $\partial_n : \mathbf{C}_n(K,G) \to \mathbf{C}_{n-1}(K,G)$ is the linear transformation defined by its action on basis vectors $\sigma = \{v_1, v_2, \ldots, v_n\} \in \mathbf{C}_n$, the formal sum:*

$$\partial_n(\{v_1, v_2, \ldots, v_n\}) = \sum_{i=1}^{n} (-1)^i \{v_1, \ldots, \hat{v}_i, v_n\}$$

where $\hat{v}_i$ indicates the omission of vertex v_i.

Definition 4. *An n-chain $\mathbf{x}$ with $\partial_n(\mathbf{x}) = 0$ is called an n-**cycle**. The set of all n-cycles forms a subspace of the chain group, the $n-$**cycle group**, denoted*

$$\mathbf{Z}_n(K) = \ker \partial_n \subset \mathbf{C}_n(K)$$

*An n-chain $\mathbf{x}$ for which there exists $n+1$-chain $\mathbf{k}$ such that $\partial_{n+1})(\mathbf{k}) = \mathbf{x}$ is called an n-**boundary**. The set of all n-boundaries forms a subspace of the chain group, the n-**boundary group**, denoted*

$$\mathbf{B}_n(K) = \mathit{Im}\, \partial_{n+1} \subset \mathbf{C}_n(K)$$

Definition 5. *The **n-th homology group of** K, $\mathbf{H}_n(K)$ is defined by*

$$\mathbf{H}_n(K) = \frac{\mathbf{Z}_n(K)}{\mathbf{B}_n(K)} = \frac{\ker \partial_n}{\mathit{Im}\, \partial_{n+1}}$$

In other words, $\mathbf{H}_n(K)$ is the set of all n-cycles which are not also n-boundaries.

Definition 6. *For a simplicial complex K, a **filtration** of K is a sequence of subcomplexes $\{K_i\}_{i \in \{1,\ldots,n\}}$ such that:*

$$K_1 \subset K_2 \subset \cdots \subset K_{n-1} \subset K_n = K$$

Remark 1. *Let $f : K \to \mathbb{R}$ such that if $\sigma_1 \subseteq \sigma_2$, $f(\sigma_1) \leq f(\sigma_2)$. Then f induces a natural filtration $\{K_{\epsilon_i}\}_{\epsilon_i \in f(K)}$, where*

$$K_{\epsilon_i} = \{\sigma \in K : f(\sigma) \leq \epsilon_i\}$$

Proof. Given $\epsilon_i \in f(K)$, any simplex $\sigma \in K_{\epsilon_i}$ and $\sigma^* \subset \sigma$, by supposition $f(\sigma^*) \leq f(\sigma)$ so that $\sigma^* \in K_{\epsilon_i}$. Then K_{ϵ_i} is a simplicial complex, and given $\epsilon_j < \epsilon_k$, if $\sigma \in K_{\epsilon_j}$, then $f(\sigma) < \epsilon_j < \epsilon_k$ so that $K_{\epsilon_j} \subset K_{\epsilon_k}$ □

In practice, it is often convenient to construct a filtration as induced by such a function f rather than explicitly specifying a sequence of nested subcomplexes of K.

Example 1. *Given a finite set of points in $X = \{\mathbf{x}_1, \ldots, \mathbf{x}_m\} \subset \mathbb{R}^n$ define the Vietoris-Rips complex of radius ϵ is given by*

$$VR_\epsilon(X) = \{\sigma \subset \mathcal{P}(X) : \{\mathbf{x}_{k_i}\}_{i\in\{1,\ldots,n\}} \in \sigma \iff d(\mathbf{x}_{k_i}, \mathbf{x}_{k_j}) < \epsilon \textit{ for all } \mathbf{x}_{k_i}, \mathbf{x}_{k_j} \in \{\mathbf{x}_{k_i}\}\}$$

In other words, $VR_\epsilon(X)$ denotes the complex composed of all $k < n$-simplices σ with vertices in X such that the distance between any two vertices in σ is at most ϵ. Then $\{VR_\epsilon(X) : \epsilon = d(x_i, x_j), x_i, x_j \in X\}$ is a valid filtration for $K = VR_{\epsilon_{max}}(X)$ where $\epsilon_{max} = \max_{i,j} d(x_i, x_j)$. The Vietoris-Rips complex and the Vietoris-Rips filtration are standard constructions to infer topological structure from point cloud data.

Definition 7. *Given a filtered simplicial complex $\{K_{\epsilon_i}\}_{i=1}^m$ and an n-chain $\mathbf{c} \in \mathbf{C}_n(K_{\epsilon_i}$ for some i, the **birth** and **death parameters** for $\mathbf{c}$ are given by:*

$$\textit{Birth}(\mathbf{c}) = \min\{\epsilon_i : \boldsymbol{c} \in \mathbf{C}_n(K_{\epsilon_i})\}$$

$$\textit{Death}(\mathbf{c}) = \begin{cases} \min\{\epsilon_i : \boldsymbol{c} \in \mathbf{B}_n(K_{\epsilon_i})\} & \exists \mathbf{k} : \mathbf{c} = \partial_{n+1}(\mathbf{k}) \\ \infty & \textit{else} \end{cases}$$

Definition 8. *The **persistent lifetime**. of $\mathbf{c} \in \mathbf{C}_n(K_{\epsilon_i})$, is denoted*

$$\mathcal{L}(\mathbf{c}) = [\textit{Birth}(\mathbf{c}), \textit{Death}(\mathbf{c}))$$

Intuitively, a chain's birth, death, and lifetime represents the duration that a homological feature is present across a filtration. In this sense, the lifetime of a homology class in some sense represents the prominence of this topological feature in the underlying data.

Methods S2: Derivation of multiscale Normalized Mutual Information, related to STAR Methods.

In this section, we derive our version of the NMI, defined in the main text's methods.
Let $\mathbf{U}^* \in [0,1]^{m\times n_1}$, $\mathbf{V}^* \in [0,1]^{m\times n_2}$ be matrices representing two sets of multiscale domains, where entries between $[0,1]$ represent coreness scores for each of m transcriptomic spots, and where $\mathbf{U}^*$ contains n_1 domains and $\mathbf{V}^*$ contains n_2 domains. In other words, each column $\mathbf{U}_1, \ldots, \mathbf{U}_{n_1}$ of $\mathbf{U}^*$ is a vector of the coreness scores for the i-th multiscale domain in $\mathbf{U}^*$.

Let $\mathbf{U}$, $\mathbf{V}$ be the row-normalized $\mathbf{U}^*$, $\mathbf{V}^*$, i.e.

$$U_{ij} = \frac{U^*_{ij}}{\sum_{k=1}^{n_1} U^*_{ik}}$$

$$V_{ij} = \frac{V^*_{ij}}{\sum_{k=1}^{n_2} V^*_{ik}}$$

Because rows of $\mathbf{U}$ sum to 1, we interpret U_{ij} as a probability that transcriptomic spot i belongs to domain $\mathbf{U}_j$.

The standard mutual information score for single-scale clusterings $\mathbf{U}$ and $\mathbf{V}$ is derived from

$$MI(\mathbf{U}, \mathbf{V}) := \sum_{i=1}^{n_1} \sum_{j=1}^{n_2} P(\mathbf{U}_i, \mathbf{V}_j) \ln \frac{P(\mathbf{U}_i, \mathbf{V}_j)}{P(\mathbf{U}_i)P(\mathbf{V}_j)} \tag{3}$$

Here, $P(\mathbf{U}_i)$ is the probability that a random spot belongs to the domain $\mathbf{U}_i$, $P(\mathbf{V}_j)$ is the probability that a random spot belongs to domain $\mathbf{V}_j$, and $P(\mathbf{U}_i, \mathbf{V}_j)$ is the probability that a random spot belongs to both $\mathbf{U}_i$ and $\mathbf{V}_j$. To obtain the multiscale NMI, we will derive each of these terms individually.

Firstly, we consider $P(\mathbf{U}_i)$. By the law of total probability, we can express the probability that a randomly chosen spot belongs to $\mathbf{U}_i$ as the sum of probabilities that spot $k \in \{1, \ldots, m\}$ is randomly drawn times the conditional probability that $k \in \mathbf{U}_i$ given k:

$$P(\mathbf{U}_i) = \sum_{k=1}^{m} P(k \in \mathbf{U}_i | k) P(k)$$

By stipulation, $P(k \in \mathbf{U}_i | k)$ is nothing but U_{ki}, while $P(k) = \frac{1}{m}$. Then

$$\begin{aligned} P(\mathbf{U}_i) &= \sum_{k=1}^{m} \frac{U_{ki}}{m} \\ &= \frac{\|\mathbf{U}_i\|_1}{m} \end{aligned} \tag{4}$$

By the same reasoning,

$$P(\mathbf{V}_j) = \frac{\|\mathbf{V}_j\|_1}{m} \tag{5}$$

Now, consider $P(\mathbf{U}_i, \mathbf{V}_j)$. Again, by the law of total probability

$$P(\mathbf{U}_i, \mathbf{V}_j) = \sum_{k=1}^{m} P(k \in \mathbf{U}_i \text{ and } k \in \mathbf{V}_j | k) P(k) = \sum_{k=1}^{m} P(k \in \mathbf{U}_i | k) P(k \in \mathbf{V}_j | k) P(k)$$

Again applying $P(k \in \mathbf{U}_i | k) = U_{ki}$ and $P(k) = \frac{1}{m}$,

$$P(\mathbf{U}_i, \mathbf{V}_j) = \sum_{k=1}^{m} \frac{U_{ki} V_{kj}}{m} = \frac{\mathbf{U}_i^\top \mathbf{V}_j}{m} \tag{6}$$

We plug the identities in equations 2-4 into equation 1, so that the mutual information score for multiscale domains is

$$MI(\mathbf{U}, \mathbf{V}) = \sum_{i=1}^{n_1} \sum_{j=1}^{n_2} \frac{\mathbf{U}_i^\top \mathbf{V}_j}{m} \ln \left[\frac{m \mathbf{U}_i^\top \mathbf{V}_j}{\|\mathbf{U}_i\|_1 \|\mathbf{V}_j\|_1} \right] \tag{7}$$

Various kinds of normalizations can be applied to the mutual information score; here, we normalize by the average entropy of $\mathbf{U}$ and $\mathbf{V}$. Shannon's entropy is defined as

$$H(\mathbf{U}) = -\sum_{i=1}^{n_1} P(\mathbf{U}_i) \ln P(\mathbf{U}_i) = -\sum_{i=1}^{n_1} \frac{\|\mathbf{U}_i\|_1}{m} \ln \left[\frac{\|\mathbf{U}_i\|_1}{m} \right] \tag{8}$$

So that

$$NMI(\mathbf{U},\mathbf{V}) = \frac{MI(\mathbf{U},\mathbf{V})}{1/2\,[H(\mathbf{U}) + H(\mathbf{V})]} \tag{9}$$

Def: For binary clusterings $\mathbf{U}, \mathbf{V}$, the standard (non-multiscale) MI and H are defined as follows.

$$MI(\mathbf{U},\mathbf{V}) = \sum_{i=1}^{n_1}\sum_{j=1}^{n_2} \frac{|\mathbf{U}_i \cap \mathbf{V}_j|}{m} \ln\left[\frac{m|\mathbf{U}_i \cap \mathbf{V}_j|}{|\mathbf{U}_i||\mathbf{V}_j|}\right] \tag{10}$$

$$H(\mathbf{U}) = -\sum_{i=1}^{n_1} \frac{|\mathbf{U}_i|}{m} \ln\left[\frac{|\mathbf{U}_i|}{m}\right] \tag{11}$$

where $| * |$ denotes the number of elements in the cluster.

Claim: In the case where $\mathbf{U}$ and $\mathbf{V}$ are sets of binary clusters, the multiscale $NMI(\mathbf{U},\mathbf{V})$ is equivalent to the standard definition.

Proof. We will show that $H(\mathbf{U})$ and $MI(\mathbf{U},\mathbf{V})$ are equivalent in both formulations, which suffices to show that $NMI(\mathbf{U},\mathbf{V})$ is too.

Consider the identity from equation 2,

$$P(\mathbf{U}_i) = \frac{\|\mathbf{U}_i\|_1}{m}$$

If $\mathbf{U}_i$ is binary, entries U_{ki} are either 0 if $k \notin \mathbf{U}_i$ and 1 if $k \in \mathbf{U}_i$. Then clearly

$$\|\mathbf{U}_i\|_1 = \sum_k U_{ki} = \sum_{k \in U_i} 1 = |\mathbf{U}_i|$$

which implies that

$$P(\mathbf{U}_i) = \frac{|\mathbf{U}_i|}{m} \tag{12}$$

In the same way,

$$P(\mathbf{V}_j) = \frac{|\mathbf{V}_j|}{m} \tag{13}$$

Now we consider the identity from equation 4,

$$P(\mathbf{U}_i, \mathbf{V}_j) = \sum_{k=1}^{m} \frac{U_{ki}V_{kj}}{m} = \frac{\mathbf{U}_i^\top \mathbf{V}_j}{m}$$

Since $\mathbf{U}_i, \mathbf{V}_j$ are binary, $U_{ki}V_{kj} = 1$ if and only if $k \in \mathbf{U}_i$ and $k \in \mathbf{V}_j$, and $U_{ki}V_{kj} = 0$ otherwise. Then

$$\begin{aligned} P(\mathbf{U}_i, \mathbf{V}_j) &= \sum_{k \in \mathbf{U}_i \cap \mathbf{V}_j} \frac{1}{m} \\ &= \frac{|\mathbf{U}_i \cap \mathbf{V}_j|}{m} \end{aligned} \tag{14}$$

We plug equations 10, 11, 12 into equation 1, giving

$$MI(\mathbf{U},\mathbf{V}) = \sum_{i=1}^{n_1}\sum_{j=1}^{n_2} \frac{|\mathbf{U}_i \cap \mathbf{V}_j|}{m} \ln\left[\frac{m|\mathbf{U}_i \cap \mathbf{V}_j|}{|\mathbf{U}_i||\mathbf{V}_j|}\right] \tag{15}$$

Similarly,

$$H(\mathbf{U}) = -\sum_{i=1}^{n_1} \frac{|\mathbf{U}_i|}{m} \ln\left[\frac{|\mathbf{U}_i|}{m}\right] \tag{16}$$

We see that equations 13 and 14 match equations 8 and 9, as desired. □

Supplemental References

1. Munkres, J. R. Topology. 2 ed. Prentice Hall, Inc. (2000). ISBN 0131816292. URL: http://www.worldcat.org/isbn/0131816292.
2. Hatcher, A. Algebraic topology. Cambridge: Cambridge University Press (2002). ISBN 0-521-79160-X; 0-521-79540-0.
3. Dey, T. K., and Wang, Y. Computational Topology for Data Analysis. Cambridge University Press (2022).
